# Supplementary material for: PARP1 exhibits enhanced association and catalytic efficiency with γH2A.X-nucleosome
Source: Nat Commun. 2019 Dec 17;10:5751. doi: 10.1038/s41467-019-13641-0 (PMC6917767; doi:10.1038/s41467-019-13641-0)
Supplement: Supplementary file 1 — Supplementary Information [file 41467_2019_13641_MOESM1_ESM.pdf]

PARP1 Exhibits Enhanced Association and Catalytic Efficiency with γH2A.X-Nucleosome

SUPPLEMENTARY INFORMATION

Deepti Sharma, Louis De Falco, Sivaraman Padavattan, Chang Rao,

Susana Geifman-Shochat, Chuan Fa Liu & Curt A. Davey

CONTENTS

15 Figures: pages 2-16

1 Table: page 17

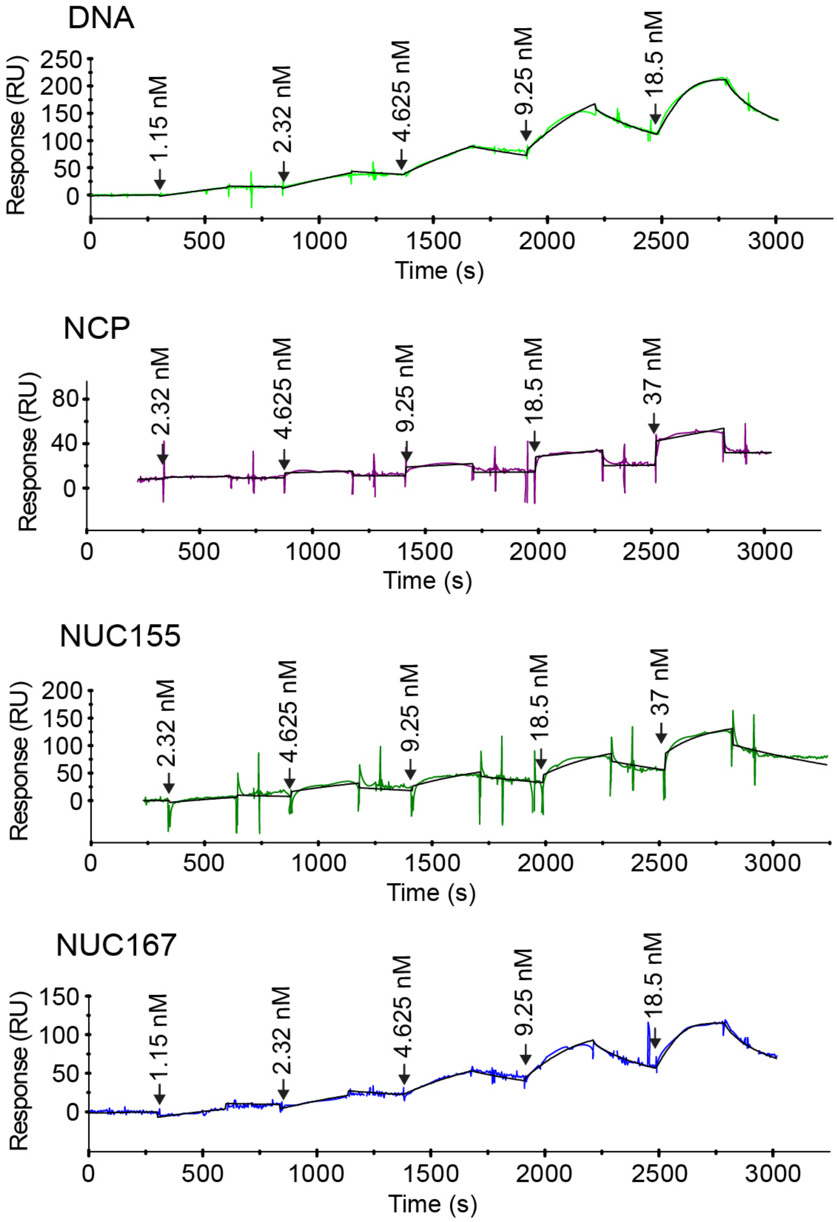

Supplementary Figure 1. SPR sensorgrams for NPARP1 interaction with four different activators. Kinetic parameters given in Fig. 1b and Supplementary Table 3.

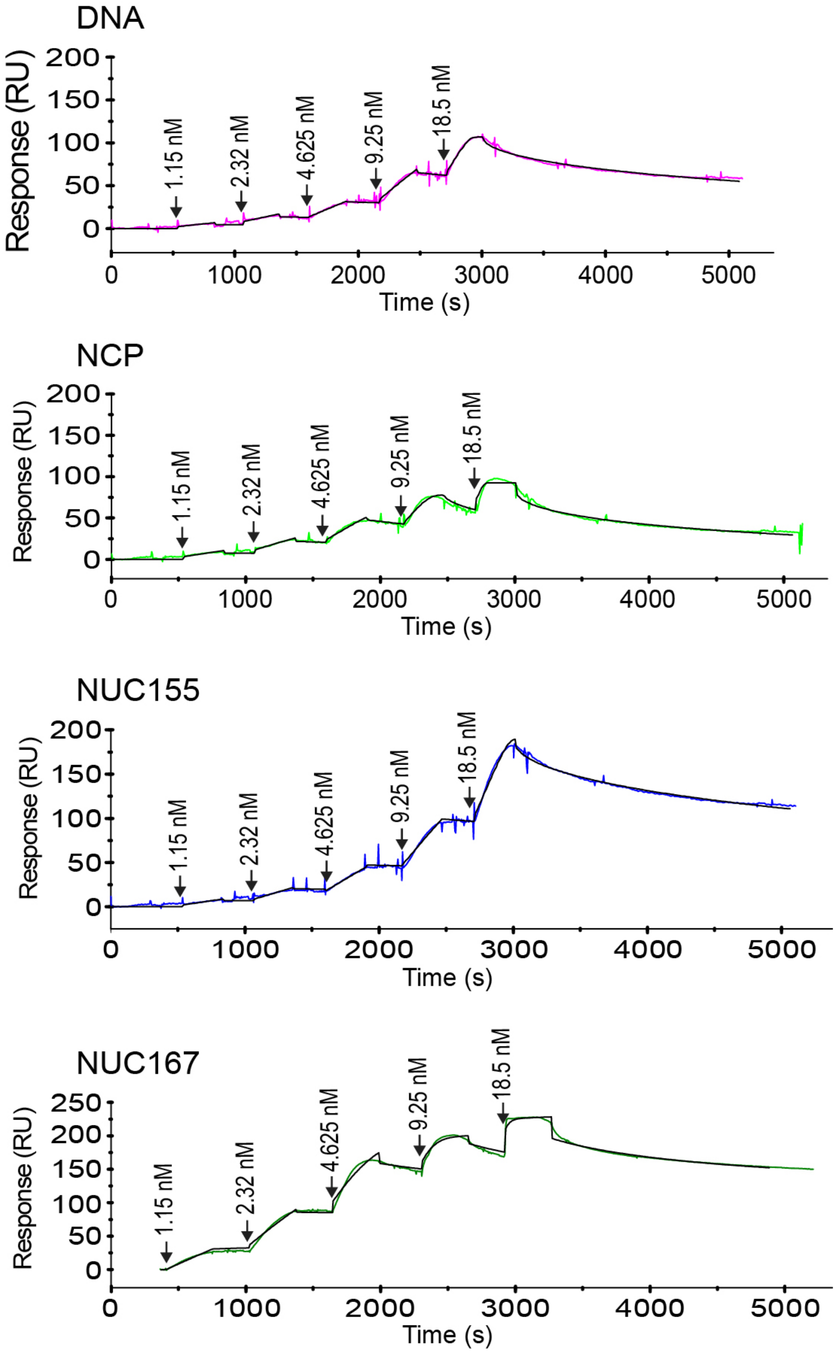

Supplementary Figure 2. SPR sensorgrams for PARP1 interaction with four different activators. Kinetic parameters given in Fig. 1b and Supplementary Table 3.

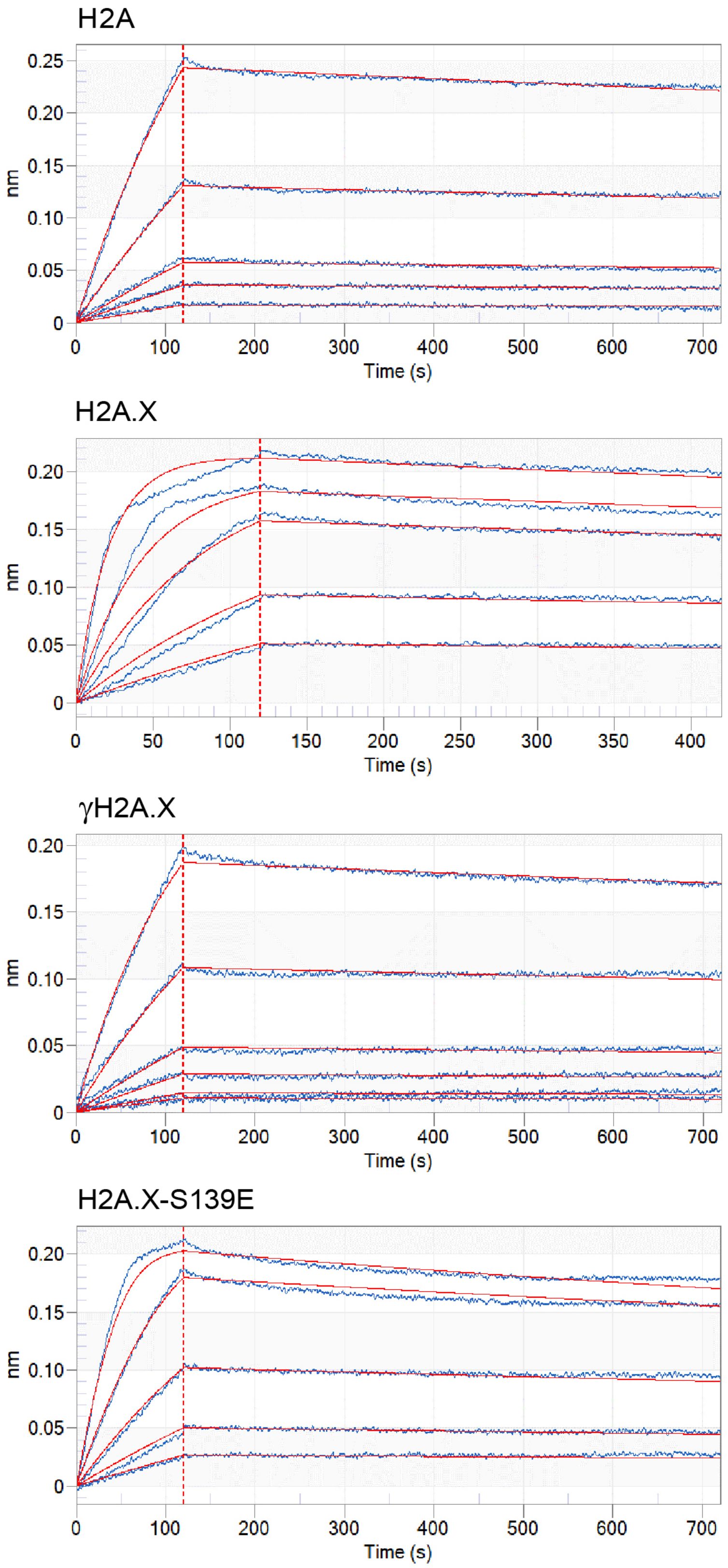

Supplementary Figure 3. BLI sensorgrams for PARP1 interaction with four different NUC167 activators. Kinetic parameters given in Fig. 1b and Supplementary Table 3.

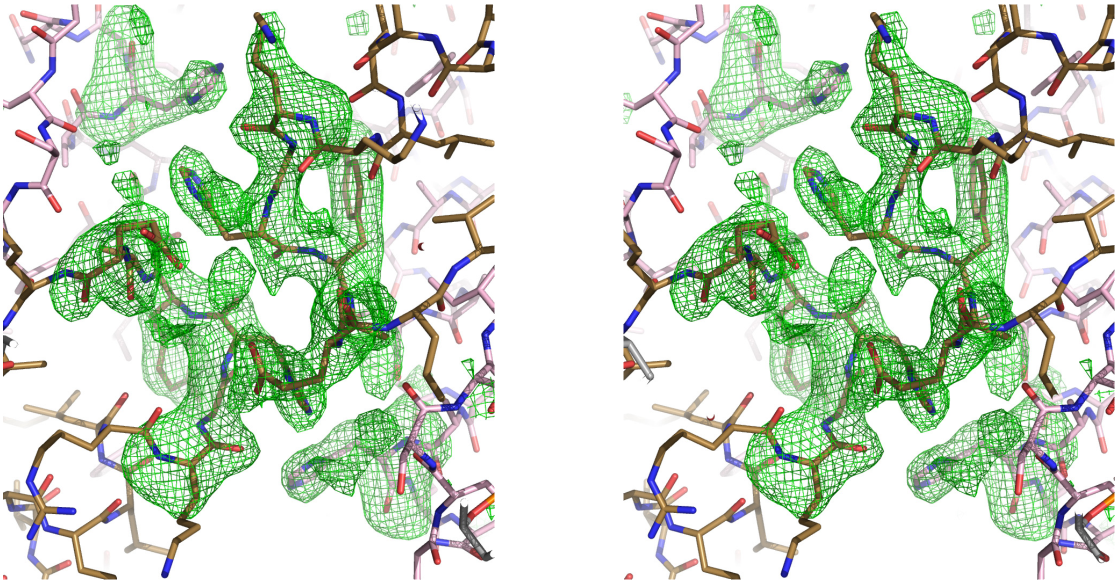

Supplementary Figure 4. Experimental electron density corresponding to the H2A.X-H2B dimer-dimer interface in the X-ray crystal structure of the H2A.X-S139E-NCP (in stereo view). An F_O-F_C omit electron density map (green; contoured at 2.5σ) are superimposed onto the refined model. The omit map was calculated with residues K36, G37, H38, Y39, A40, E41 of H2A.X (bronze carbon atoms) and residue H79 of H2B (pink carbon atoms) omitted from the model.

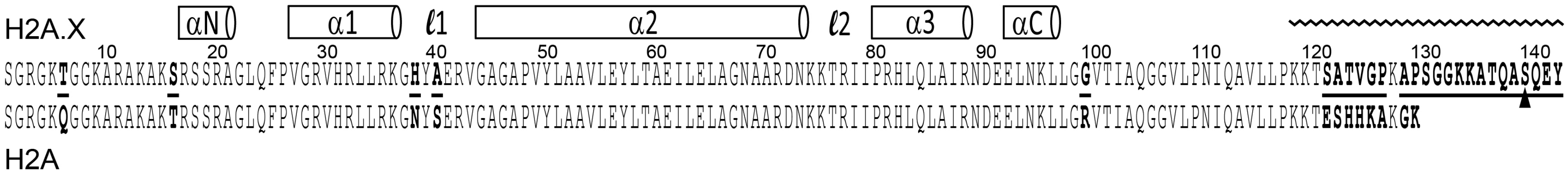

Supplementary Figure 5. Sequence comparison of *H. sapiens* H2A.X with H2A (subtype 1). Differing residues are indicated with a bar, while the site of phosphorylation, yielding γH2A.X, is designated with an arrowhead. Secondary structure motifs of the histone fold and extensions (α, α-helix; ***ℓ***, loop) are shown at top, with the wavy line indicating the C-terminal tail, which is partially or completely disordered (P117 is the last residue that interacts in a defined fashion with other histone elements). Note that residue 40 is not conserved across H2A subtypes.

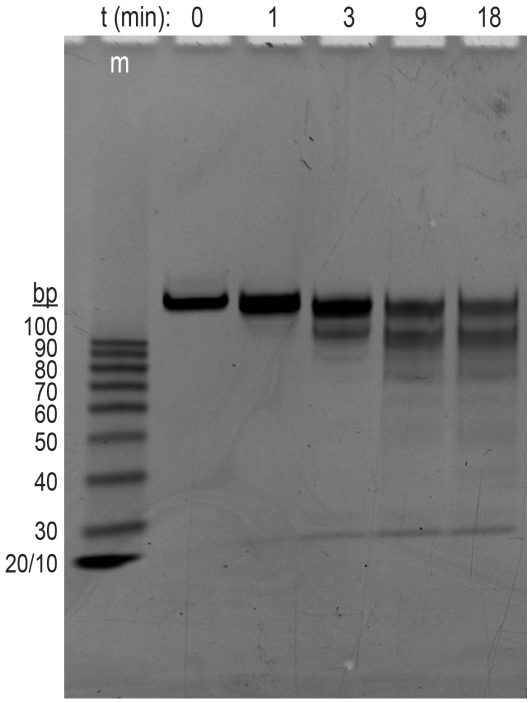

Supplementary Figure 6. Micrococcal nuclease digestion analysis of H2A.X-NCP. After incubation with nuclease for the specified times, samples were visualized by native DNA PAGE subsequent to degradation of proteins (m, 10 bp DNA ladder).

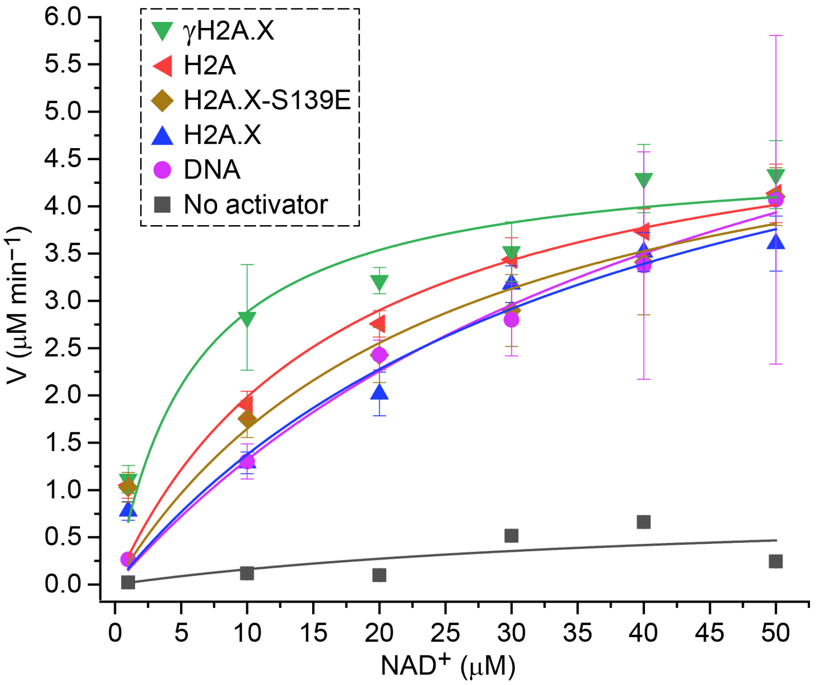

Supplementary Figure 7. Influence of activator binding on PARP1 catalytic activity. Catalytic profile for PARP1 activation by H2A- and H2A.X-nucleosomes (as shown in Fig. 5a) and by naked DNA. Catalytic parameters for the DNA are 7.8±1.2 μM min^{-1} (V_{max}), 49.1±13.5 μM (K_M), 1131.3 min^{-1} (k_{cat}) and 23.1 min^{-1} μM^{-1} (k_{cat}/K_M; values for the other activators are given in Fig. 5a; mean±s.d., n=2 independent experiments).

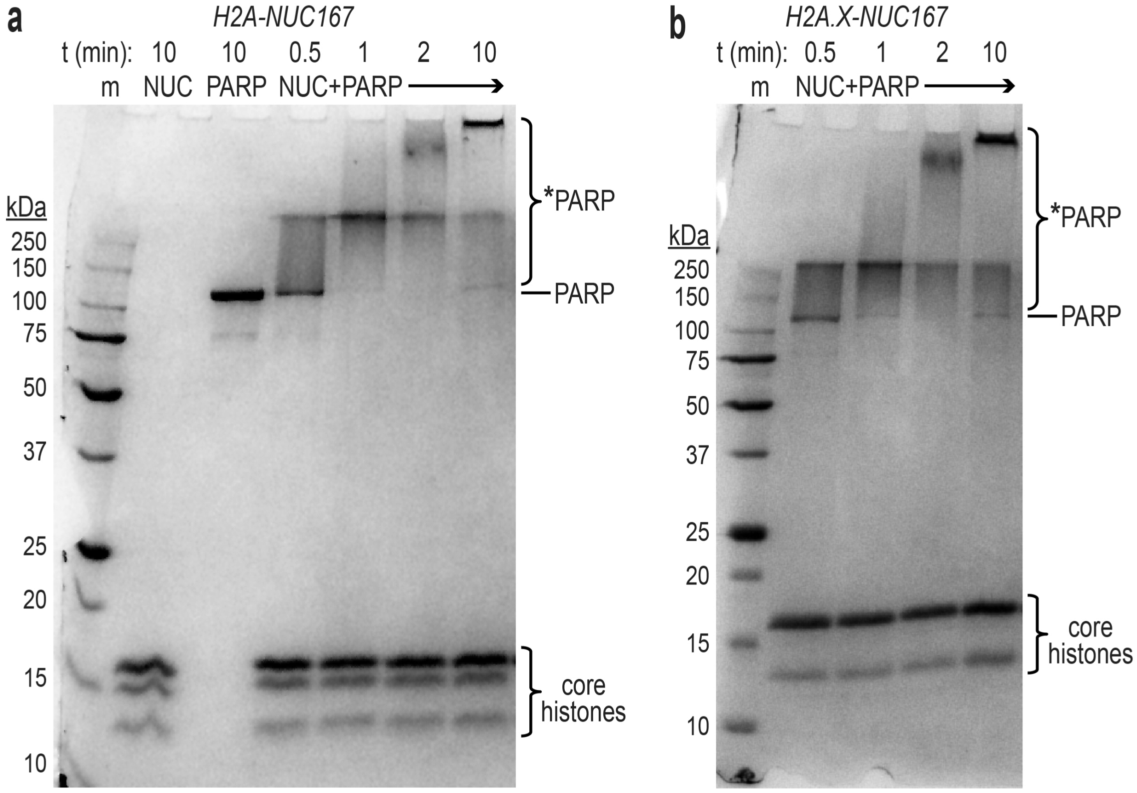

Supplementary Figure 8. Auto-PARylation of PARP1 observed with activation by NUC167 in the presence of NAD^+. (**a**,**b**) Samples were incubated with 5 mM NAD^+ for the specified time prior to SDS-PAGE analysis (protein molecular weight marker, m). NUC-containing samples consist of NUC167 assembled with either H2A (**a**) or H2A.X (**b**). Cumulative PARylation is observed, which is only on PARP1 itself (*PARP).

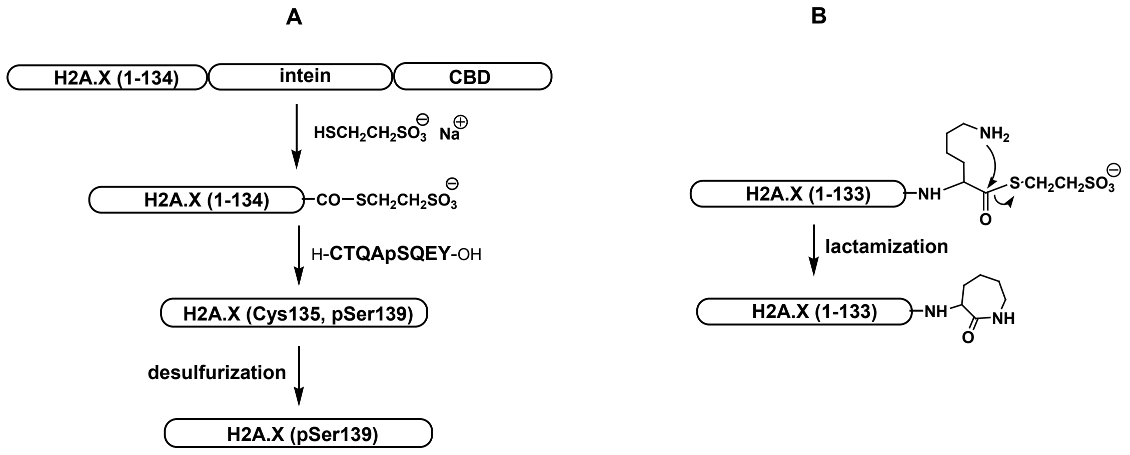

Supplementary Figure 9. Overview of method for synthesis of γH2A.X [H2A.X (pS139)]. **a**) Scheme of the overall synthetic strategy. **b**) Side reaction that leads to the C-terminal lactam product of H2A.X (1-134).

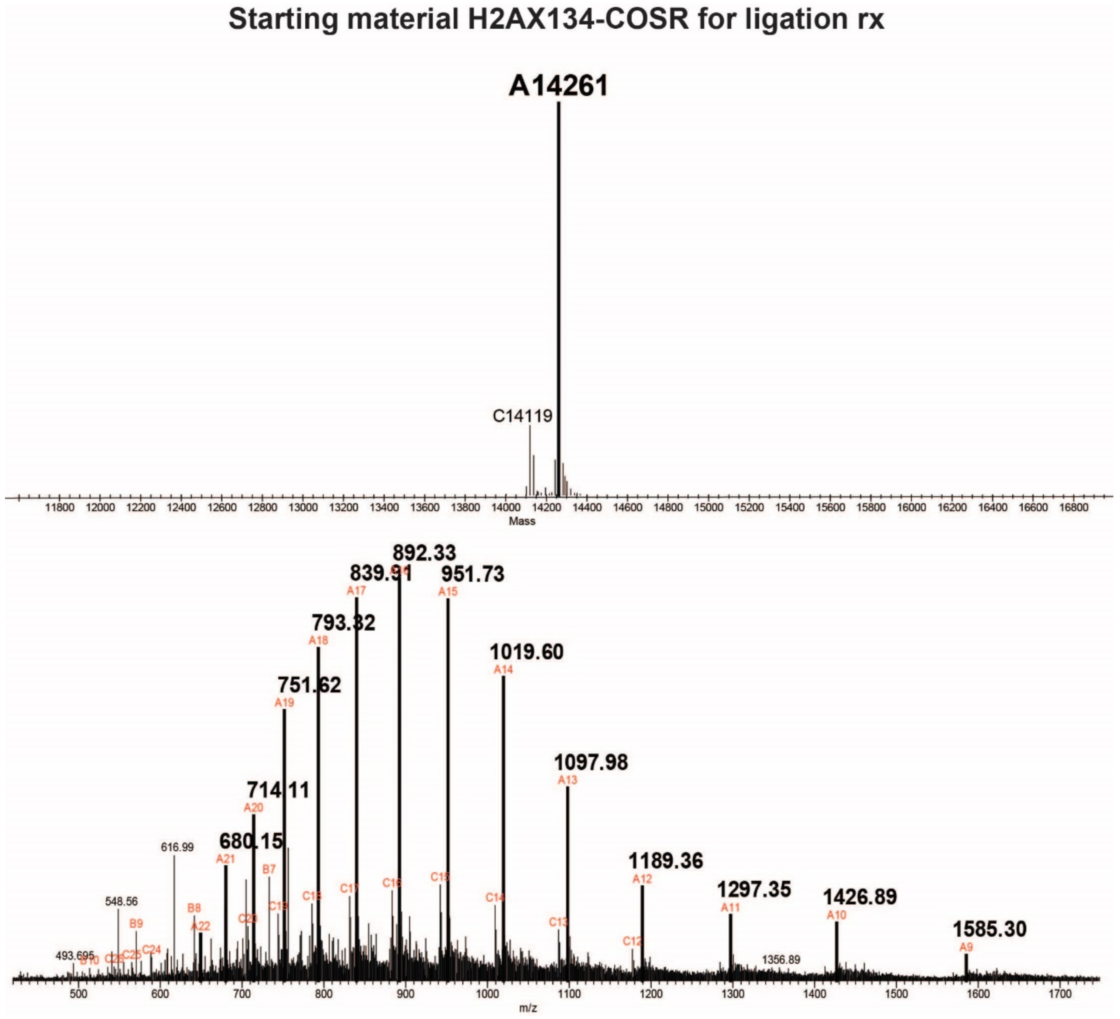

Supplementary Figure 10. Monitoring of the preparation of the H2A.X (Cys135, pSer139) intermediate via ligation by mass spectrometry analysis. ESI-MS of the ligation starting material, the MES thioester of H2A.X (1-134). *m/z* $[M+H]^+$ found: 14,261, calcd: 14,259.5. The minor peak at *m/z* 14,119 corresponds to the C-terminal lactam side product of H2A.X (1-134).

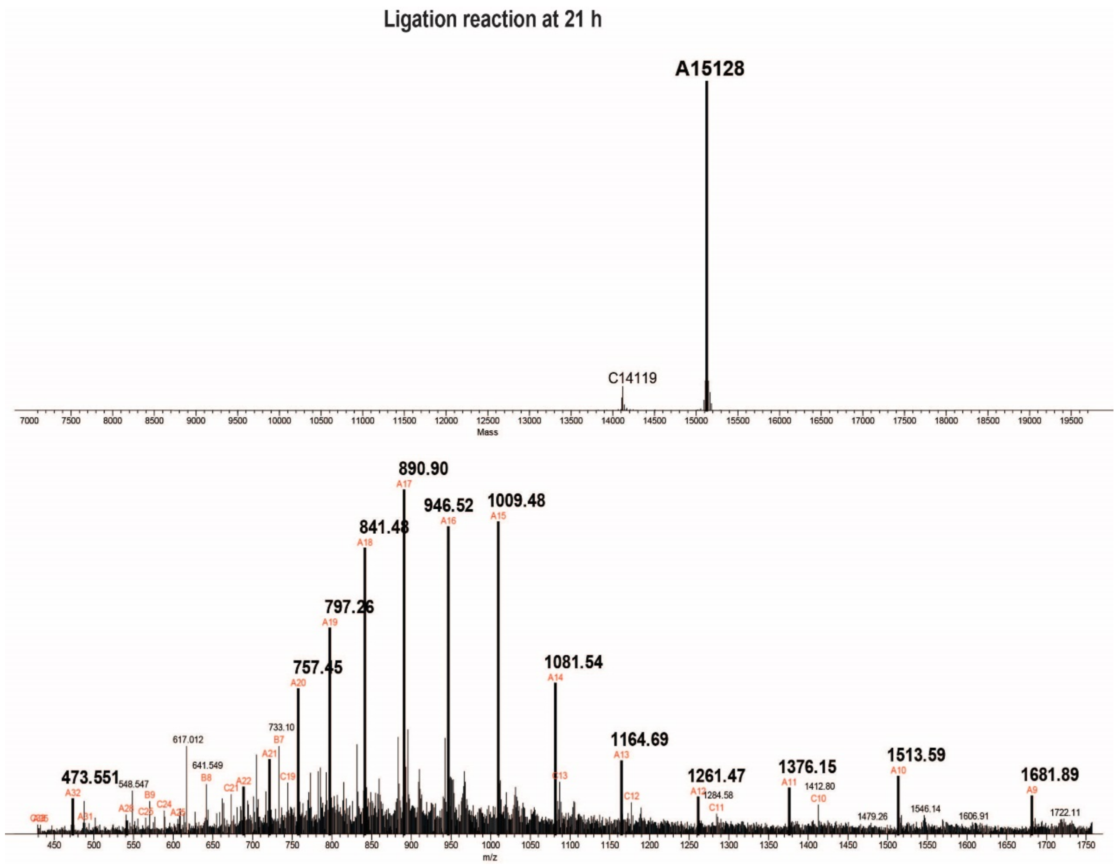

Supplementary Figure 11. Monitoring of the preparation of the H2A.X (Cys135, pSer139) intermediate via ligation by mass spectrometry analysis. ESI-MS monitoring of the ligation reaction at 21 h. The desired ligation product is H2A.X (A135C, pSer139), with *m/z* $[M+H]^+$ found: 15,128, calcd: 15,126.3. The minor peak at *m/z* 14,119 corresponds to the C-terminal lactam side product of H2A.X (1-134).

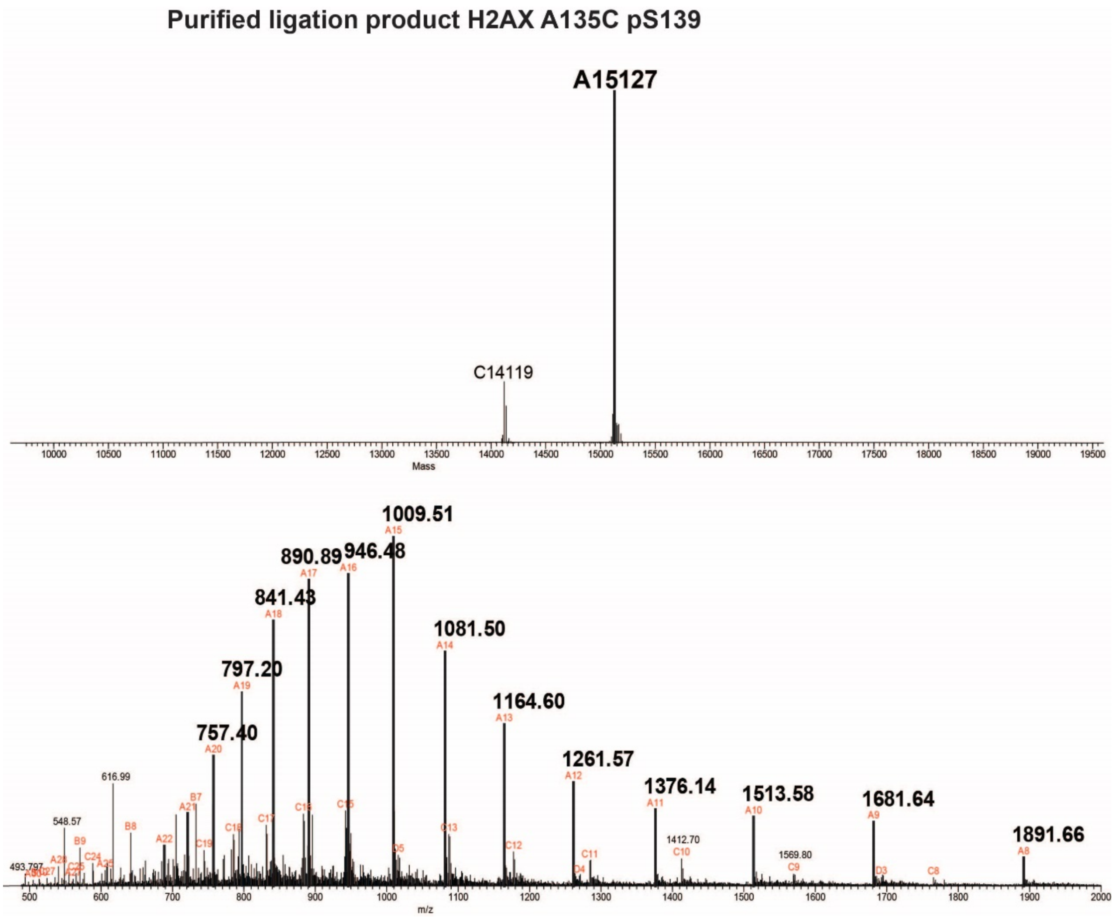

Supplementary Figure 12. Monitoring of the preparation of the H2A.X (Cys135, pSer139) intermediate via ligation by mass spectrometry analysis. ESI-MS of the purified ligation product H2A.X (A135C, pSer139), with *m/z* $[M+H]^+$ found: 15,127, calcd: 15,126.3. The minor peak at *m/z* 14,119 corresponds to the C-terminal lactam side product of H2A.X (1-134).

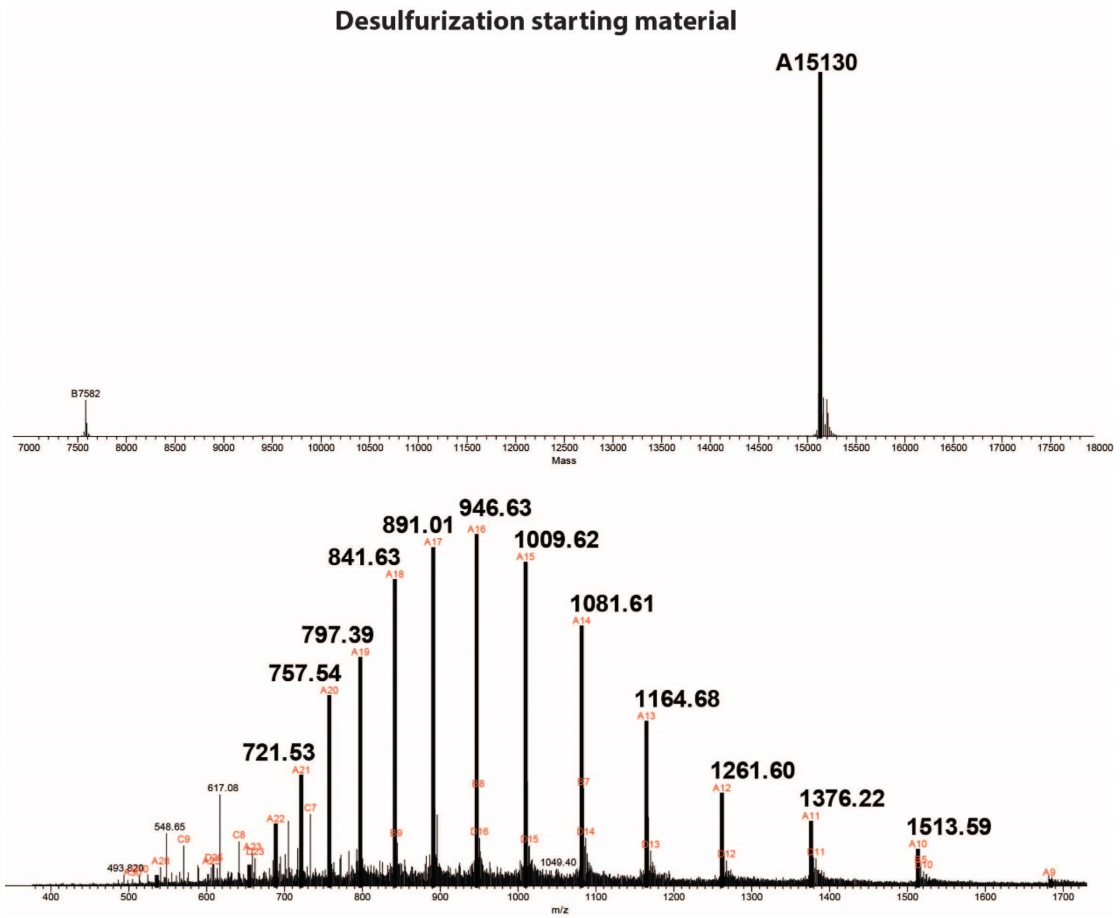

Supplementary Figure 13. Monitoring of the desulfurization of the H2A.X (Cys135, pSer139) intermediate by mass spectrometry analysis. ESI-MS of the starting material for desulfurization, H2A.X (Cys135, pSer139), after removal of the minor lactam side product.

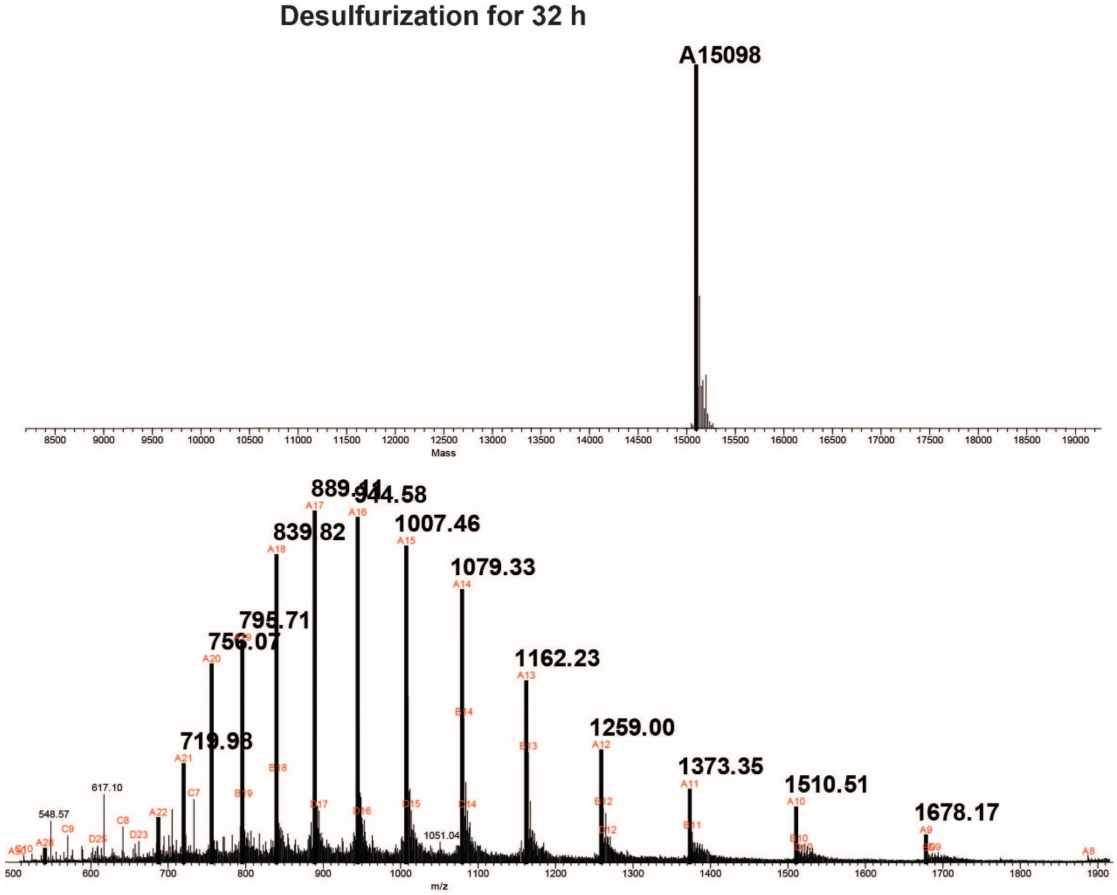

Supplementary Figure 14. Monitoring of the desulfurization of the H2A.X (Cys135, pSer139) intermediate by mass spectrometry analysis. Monitoring of the desulfurization reaction after 32 h by ESI mass spectrometry analysis.

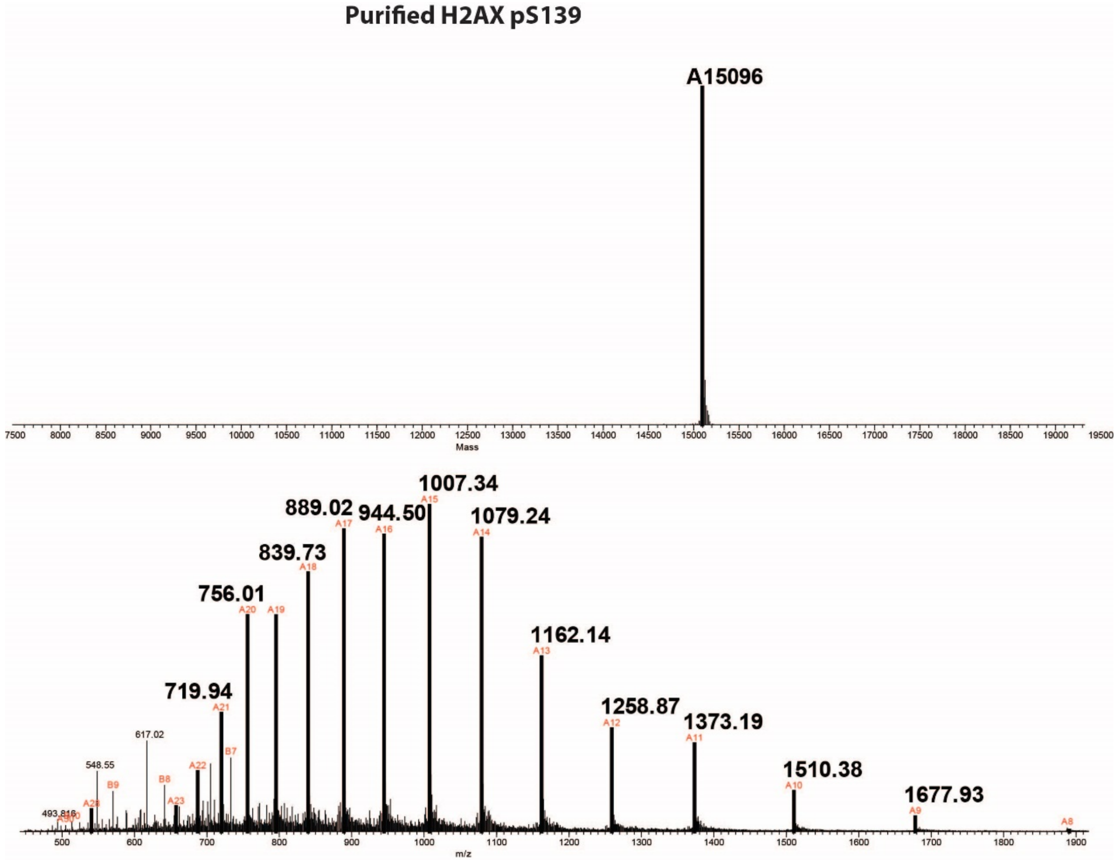

Supplementary Figure 15. Monitoring of the desulfurization of the H2A.X (Cys135, pSer139) intermediate by mass spectrometry analysis. ESI-MS of the purified final product H2A.X (pSer139) after desulfurization of H2A.X (Cys135, pSer139). *m/z* $[M+H]^+$ found: 15,096, calcd: 15,094.3.

Supplementary Table 1. Kinetic parameters for NPARP1 and PARP1 binding to naked DNA, NCP and nucleosomal substrates.

System	PARP	Activator	k_a (s^{-1} M^{-1})	k_d (s^{-1})	K_D (M)
SPR	NPARP1	DNA	3.95±1.97 E+06	2.68±1.37 E−02	6.78±4.84 E−09
SPR	NPARP1	NCP	2.26±0.03 E+04	4.24±1.08 E−03	1.87±4.78 E−07
SPR	NPARP1	NUC155	6.19±0.29 E+04	1.07±0.01 E−03	1.73±0.08 E−08
SPR	NPARP1	NUC167	3.00±2.40 E+06	2.44±2.08 E−02	8.13±9.49 E−09
SPR	PARP1	DNA	3.08±1.42 E+06	1.95±0.85 E−03	6.38±0.20 E−10
SPR	PARP1	NCP	1.06±0.24 E+06	7.43±2.18 E−04	7.01±2.59 E−10
SPR	PARP1	NUC155	1.93±0.13 E+06	1.07±0.01 E−03	5.54±0.37 E−10
SPR	PARP1	NUC167	2.28±0.03 E+06	5.62±0.07 E−04	2.46±0.03 E−10
BLI	PARP1	NUC167	8.25±0.12 E+05	1.58±0.02 E−04	1.91±0.03 E−10
BLI	PARP1	H2A.X	3.75±0.04 E+06	2.74±0.01 E−04	7.32±0.03 E−11
BLI	PARP1	γH2A.X	2.43±0.03 E+06	1.16±0.01 E−04	4.78±0.01 E−11
BLI	PARP1	H2A.X-S139E	7.50±0.13 E+06	3.28±0.05 E−04	4.38±0.01 E−11

NCP/nucleosomal activators assembled with H2A, unless otherwise indicated (last 3). H2A.X-nucleosomes (last 3) coincide with the NUC167 construct (SPR samples, mean±s.d., n=2 independent experiments; BLI samples, mean±s.d., n=3,2,3,2 independent experiments).
